# Synaptic activations of neuronal populations in the thalamocortical loop from LFP using kCSD and ICA

**DOI:** 10.1186/1471-2202-13-S1-P11

**Published:** 2012-07-16

**Authors:** Szymon Łęski, Helena Głąbska, Jan Potworowski, Daniel K Wójcik

**Affiliations:** 1 Department of Neurophysiology, Nencki Institute of Experimental Biology, Warsaw, 02-093, Poland

## 

Local field potentials (LFP), the low-frequency part of the electric potential recorded extracellularly in the brain, is a well-established measure of neural activity at the population level. However, the origin and the nature of the LFP make it harder to interpret than other electrophysiological signals such as spikes. Two problems intertwine here: first, the extracellular field spreads over significant distances from the generating population; second, the recorded signal is typically a combination of contributions from several sources. The former problem is often addressed by reconstructing the underlying currents using Current Source Density (CSD) analysis. To solve the latter issue we have employed in the past the combination of CSD and Independent Component Analysis (ICA) [1].

Here we test the combination of CSD analysis [2] and ICA on simulated LFP data obtained from a computational model of a single thalamocortical column [3,4]. We test how the components obtained by analyzing the full LFP are related to the ‘building blocks’ of population activity, that is the specific synaptic activations of individual populations of neurons. We show which populations can possibly be recorded with a limited number of electrodes and in the presence of noise, and discuss the limitations of the approach and ways to improve the reconstructions.

## References

[B1] Łęski S Kublik E Świejkowski DA Wróbel A Wójcik DK Extracting functional components of neural dynamics with Independent Component Analysis and inverse Current Source Density J Comput Neurosci 2010 29 459 473 10.1007/s10827-009-0203-1 20033271

[B2] Potworowski J Jakuczun W Łęski S Wójcik DK Kernel current source density method Neural Comput 2012 24 541 575 10.1162/NECO_a_00236 22091662

[B3] Traub RD Contreras D Cunningham MO Murray H LeBeau FEN Roopun A Bibbig A Wilent WB Higley MJ Whittington MA Single-Column Thalamocortical Network Model Exhibiting Gamma Oscillations, Sleep Spindles, and Epileptogenic Bursts J Neurophysiol 2005 93 2194 2232 15525801 10.1152/jn.00983.2004

[B4] http://senselab.med.yale.edu/ModelDb/ShowModel.asp?model=82894

